# Emodin and Aloe-Emodin Suppress Breast Cancer Cell Proliferation through ER****α**** Inhibition

**DOI:** 10.1155/2013/376123

**Published:** 2013-06-24

**Authors:** Pao-Hsuan Huang, Chih-Yang Huang, Mei-Chih Chen, Yueh-Tsung Lee, Chia-Herng Yue, Hsin-Yi Wang, Ho Lin

**Affiliations:** ^1^Department of Life Sciences, National Chung Hsing University, Taichung 40227, Taiwan; ^2^Department of Health and Nutrition Biotechnology, Asia University, Taichung 41354, Taiwan; ^3^Graduate Institute of Basic Medical Science, China Medical University, Taichung 40402, Taiwan; ^4^Department of Urology, University of Texas Southwestern Medical Center, Dallas, TX 75390, USA; ^5^Department of Surgery, Chang Bing Show Chwan Memorial Hospital, Changhua 50544, Taiwan; ^6^Department of Surgery, Tungs' Taichung MetroHarbor Hospital, Taichung 43304, Taiwan; ^7^Department of Nuclear Medicine, Taichung Veterans General Hospital, Taichung 40705, Taiwan; ^8^Department of Agricultural Biotechnology Center, National Chung Hsing University, Taichung 40227, Taiwan; ^9^Graduate Institute of Rehabilitation Science, China Medical University, Taichung 40402, Taiwan

## Abstract

The anthraquinones emodin and aloe-emodin are abundant in rhubarb. Several lines of evidence indicate that emodin and aloe-emodin have estrogenic activity as phytoestrogens. However, their effects on estrogen receptor **α** (ER**α**) activation and breast cancer cell growth remain controversial. The goal of this study is to investigate the effects and molecular mechanisms of emodin and aloe-emodin on breast cancer cell proliferation. Our results indicate that both emodin and aloe-emodin are capable of inhibiting breast cancer cell proliferation by downregulating ER**α** protein levels, thereby suppressing ER**α** transcriptional activation. Furthermore, aloe-emodin treatment led to the dissociation of heat shock protein 90 (HSP90) and ER**α** and increased ER**α** ubiquitination. Although emodin had similar effects to aloe-emodin, it was not capable of promoting HSP90/ER**α** dissociation and ER**α** ubiquitination. Protein fractionation results suggest that aloe-emodin tended to induce cytosolic ER**α** degradation. Although emodin might induce cytosolic ER**α** degradation, it primarily affected nuclear ER**α** distribution similar to the action of estrogen when protein degradation was blocked. In conclusion, our data demonstrate that emodin and aloe-emodin specifically suppress breast cancer cell proliferation by targeting ER**α** protein stability through distinct mechanisms. These findings suggest a possible application of anthraquinones in preventing or treating breast cancer in the future.

## 1. Introduction

Many phytochemicals derived from plants, including anthraquinone, have been reported to have anticancer potential. The anthraquinone derivatives emodin (1,3,8-trihydroxy-6-methylanthraquinone) and aloe-emodin (1,8-dihydroxy-3-hydroxyl-methylanthraquinone) are the main bioactive components of rhubarb (*Rheum palmatum*), which has been used in traditional Chinese medicine [1]. Aloe-emodin is also abundant in the leaves of the common plant *Aloe vera *[2]. Emodin has been widely investigated for its antibacterial [3], anti-inflammatory [4], and antiproliferative effects in several types of cancer [1]. Notably, emodin may downregulate androgen receptor (AR) and lead to the inhibition of prostate cancer cell growth [5], suggesting that anthraquinone derivatives might modulate steroid receptor activity. Several lines of evidence indicate that emodin and aloe-emodin have estrogenic activity and modulate breast cancer cell proliferation as phytoestrogen compounds [6, 7]. However, the pharmacological effects and molecular mechanisms of emodin and aloe-emodin in estrogen receptor *α* (ER*α*) modulation and breast cancer cell growth remain elusive.

 Breast cancer is a common malignancy with high lethality in women. Because ER*α* activation plays an important role in the initiation, development, and progression of breast cancer, estrogen replacement therapy is the most common strategy to suppress breast cancer progression [8]. By mimicking the structure of estrogen, synthetic estrogen-like compounds are used to compete for the binding of endogenous estrogen with ER*α* and therefore inhibit ER*α*-dependent growth of breast cancer cells [9, 10]. However, synthetic estrogens have side effects that increase the risk of cancer development due to unselective estrogenic action [11]. Although the potency of natural phytoestrogens is generally lower than that of synthetic estrogens in terms of estrogenic action, natural phytoestrogens are relatively safer with fewer side effects [12]. Therefore, studies investigating the effects and molecular mechanisms of natural herbal medicines that contain phytoestrogens as potential treatments to breast cancer are of interest.

We found that the inhibition of proliferation by emodin and aloe-emodin was ER*α*-dependent in breast cancer cell lines. Importantly, aloe-emodin treatment promotes ER*α* protein degradation by repressing the association of ER*α* and heat shock protein 90 (HSP90). Moreover, the dissociated ER*α* is ubiquitinated and targeted for proteasome-dependent degradation in the cytosol. The findings for aloe-emodin are distinct from those for emodin. Based on the above observations, these two anthraquinones could potentially be used as specific phytoestrogens to treat breast cancer.

## 2. Materials and Methods

### 2.1. Cell Lines and Cell Culture

 The human breast cancer cell lines MCF-7 and MDA-MB-453 were obtained from the Bioresource Collection and Research Center (BCRC), Food Industry Research and Development Institute, Taiwan. MCF-7 cells were grown in minimum essential medium (Gibco, Carlsbad, CA, USA) containing 10% fetal bovine serum (Gibco), 1.5 g/L NaHCO_3_, 0.1 mM nonessential amino acids (Gibco), 1 mM sodium pyruvate (Gibco), and 1% penicillin-streptomycin (Sigma, St. Louis, MO, USA). MDA-MB-453 cells were maintained in Dulbecco's modified Eagle's medium (Gibco) supplemented with 10% fetal bovine serum, 1.5 g/L NaHCO_3_, and 1% penicillin-streptomycin. All cells were incubated at 37°C in a humidified atmosphere with 5% CO_2_.

### 2.2. Cell Viability Assay

Cells were incubated for 24 hours after attachment. Cell numbers were calculated by direct counting of cells, excluding cells that stained positive for 0.2% trypan blue stain (Sigma, St. Louis, MO, USA) [13]. Cells were treated with different concentrations of aloe-emodin or emodin (ChromaDex, Irvine, CA, USA) for the indicated number of days, and then, the 3-(4,5-dimethylthiazol-2-yl)-2, 5-diphenyltetrazolium bromide (MTT, Sigma, St. Louis, MO, USA) assay was used to quantify cell proliferation. The MTT stock solution (5 mg/mL) was diluted to 0.5 mg/mL with complete culture medium, and 0.1 mL was added to each well. The yellow MTT was converted to blue formazan by living cells, a reaction that is dependent on mitochondrial enzyme activity. After using DMSO to dissolve the blue formazan, the absorbance of converted MTT could be measured at 570 nm *λ* [14].

### 2.3. Cell Fractionation and Western Blot Analysis

Cells were collected using a rubber scraper and homogenized with Na_3_VO_4_ diluted in PBS (1 : 100). After centrifugation, cells were resuspended with lysis buffer as previously described [15–19], and protease inhibitor cocktail (Roche Applied Science, Mannheim, Germany) was added, followed by incubation on ice for 45 minutes. The cell lysate was centrifuged, and the supernatant was collected as total protein extract. The protein extract was mixed with sample buffer and boiled for 10 minutes. Then, western blotting was performed as previously described [19, 20]. Briefly, SDS-polyacrylamide gel electrophoresis (SDS-PAGE) was performed, and proteins were transferred from the SDS-PAGE gel onto a polyvinylidene fluoride (PVDF) membrane. Primary antibodies were incubated with the membrane overnight, and horseradish peroxidase- (HRP-) conjugated secondary antibodies (Jackson ImmunoResearch Laboratory, West Grove, PA, USA) were applied. The ECL (western lighting chemiluminescence reagent plus, PerkinElmer Life Sciences, Shelton, CT, USA) reaction was performed, and the membranes were exposed to X-ray films to visualize protein staining (Fujifilm, Tokyo, Japan). Antibodies directed against the following proteins were used in this study: poly(ADP-ribose) polymerase (PARP, 06-557, Upstate Biotechnology, Lake Placid, NY, USA), *α*-tubulin (05-829, Upstate Biotechnology), ER*α* (sc-543 and sc-8005, Santa Cruz Biotechnology, Santa Cruz, CA, USA), cyclin D1 (sc-20044, Santa Cruz Biotechnology), HSP 90 *α*/*β* (sc-59577, Santa Cruz Biotechnology), ubiquitin (sc-8017, Santa Cruz Biotechnology), and *β*-actin (MAB1501, Millipore, Temecula, CA, USA). The quantification software used was MCID Image Analysis Evaluation.

### 2.4. Immunoprecipitation

 Cell lysate was extracted in lysis buffer, and immunoprecipitation was performed as previously described [19]. Briefly, the beads/antibody precipitated complex was prepared by mixing Protein G Mag Sepharose Xtra beads (GE Healthcare, Waukesha, WI, USA) and specific antibody at 10 *μ*g : 1 *μ*g for 2 h at room temperature. Cell lysates were mixed with beads/antibody for 12 h at 4°C, and protein was isolated by precipitation under magnetic attraction with three rounds of  PBST washes. The precipitated proteins were analyzed by western blotting after denaturation following dilution in sample buffer and boiling for 10 minutes.

### 2.5. Quantitative Real-Time PCR

 Total RNA was extracted from cells using a Miniprep Purification Kit (Genemark, Taipei, Taiwan), and reverse transcription-PCR was performed using a High-Capacity cDNA Reverse Transcription Kit (Applied Biosystems, Foster City, CA, USA) following the standard procedures recommended by the manufacturer. For reverse transcription, 2 *μ*g of total RNA was used as the first-strand cDNA template for the subsequent amplification procedure. The following primers were used to amplify the cDNAs: ER*α* (5′-TGGAGATCTTCGACATGCTG-3′ and 5′-TCCAGAGACTTCAGGGTGCT-3′) [21] and **β*-actin* (5′-TTGCCGACAGGATGCAGAA-3′ and 5′-GCCGATCCACACGGAGTACT-3′). cDNA and primers were mixed within FastStart Universal SYBR Green Master (Roche Applied Science) and measured using a real-time PCR instrument (Applied Biosystems). Data presented by Ct values were analyzed and adjusted relative to levels of the **β*-actin* house-keeping gene.

### 2.6. Transfection and Reporter Assays

Cells were plated for at least 24 h and had reached 80% confluency prior to transfection. Expression plasmid was premixed within Lipofectamine 2000 Reagent (Invitrogen, Carlsbad, CA, USA) according to the manufacturer's instructions. The liposome/plasmid complex was transfected into MCF-7 cells incubated with Opti-MEM (Gibco) for 6 h, and then culture medium was added for exogenous protein expression. The pCMV5-ER*α* expression plasmid was kindly provided by Professor Chih-Yang Huang, Graduate Institute of Basic Medical Science, China Medical University, Taichung, Taiwan. A reporter assay was performed to detected ER*α* transcriptional activity after cells were transfected with 3 × ERE-containing-promoter luciferase reporter gene (pGL2-TK-3 × ERE, gift from Dr. Chih-Yang Huang, China Medical University, Taichung, Taiwan) and internal control pSV-*β*-galactosidase expression plasmid (gift from Dr. Jeremy J. W. Chen, National Chuang Hsing University, Taichung, Taiwan). Reporter gene luciferase production was performed using the Dual-Light System (Applied Biosystems), and measurements were performed using a 1420 Multilabel Counter VICTOR^3^ instrument (PerkinElmer Life Sciences). The raw data were normalized to *β*-galactosidase activity to control for varying transfection efficiencies [19].

### 2.7. Statistics

 All values are presented as the mean ± standard error of the mean (SEM). In all cases, Student's *t*-test was used to assess the results of cell proliferation and reporter assays. A difference between two means was considered statistically significant when *P* < 0.05.

## 3. Results

### 3.1. ER*α* Is Important for the Growth Inhibition Induced by Emodin and Aloe-Emodin

**Figure 1:**
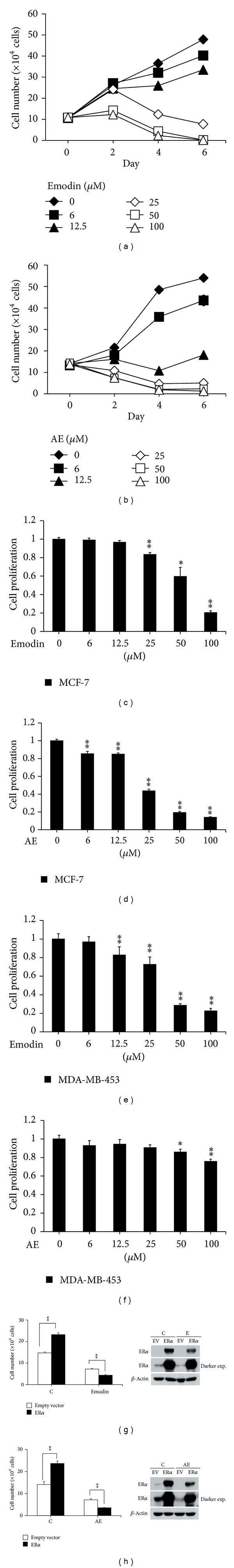
Emodin and aloe-emodin inhibit breast cancer cell growth. MCF-7 cells were treated with various concentrations of emodin (a) or aloe-emodin (b) for 0 to 6 days. Cell numbers were calculated by trypan blue staining. (c–f) MCF-7 and MDA-MB-453 cells were treated with various concentrations of emodin or aloe-emodin for 4 days. Cell proliferation was determined by an MTT assay. The control cell proliferation rate was set at 1. MCF-7 cells were transiently transfected with empty vector or pCMV5-ER*α* and treated with 25 *μ*M emodin (g) or aloe-emodin (h) for 4 days. The transfected efficiencies of ER*α* overexpression and cell numbers were detected by western blotting and trypan blue staining, respectively. The results were presented as the mean ± SEM. * and ** correspond to *P* < 0.05 and *P* < 0.01, respectively, versus the control group or empty vector group.

 The effects of different concentrations (0, 6, 12.5, 25, 50, or 100 *μ*M) of emodin and aloe-emodin on the growth of the ER*α*-positive breast cancer cell line MCF-7 were determined by cell number counting (0–6 days) and MTT assays (4 days). Emodin and aloe-emodin treatment led to dose-dependent suppression of MCF-7 growth (Figures 1(a) and 1(b)). Notably, 12.5 *μ*M or higher concentrations of aloe-emodin had stronger effects on MCF-7 growth compared to the same dosage of emodin. MTT assays showed significant inhibition of MCF-7 proliferation in a dose-dependent manner following emodin treatment at concentrations of 25 to 100 *μ*M (Figure 1(c)) and of aloe-emodin treatment at concentrations of 6 to 100 *μ*M (Figure 1(d)). Whereas emodin was more effective against the ER*α*-negative breast cancer cell line MDA-MB-453 than against MCF-7 (Figure 1(e)), the inhibitory effects of aloe-emodin on the growth of MDA-MB-453 was moderate compared to the effects on MCF-7 cells (Figure 1(f)). In which, 25 *μ*M of aloe-emodin was not able to affect MDA-MB-453 cell proliferation (Figure 1(f)), while 25 *μ*M of emodin significantly reduced that cell proliferation (Figure 1(e)) implies that the effects of aloe-emodin might be associated with ER*α* and distinct to emodin. To further investigate whether ER*α* was involved in the inhibitory effects of the emodin and aloe-emodin treatments, the effects of emodin and aloe-emodin on the growth of MCF-7 with or without ER*α* overexpression were investigated. Following a 25 *μ*M treatment with emodin (Figure 1(g)) or aloe-emodin (Figure 1(h)), the ER*α*-overexpressing cells were more sensitive to drug treatments compared to control cells. Similar results were also observed in another ER*α*-positive cell line, T47D (data not shown). These data indicate that ER*α* protein plays an important role in the emodin- and aloe-emodin-induced suppression of breast cancer cell proliferation, although the potency of these two compounds are slightly different.

### 3.2. Emodin and Aloe-Emodin Decrease ER*α* Protein Levels in a Time- and Dose-Dependent Manner

**Figure 2:**
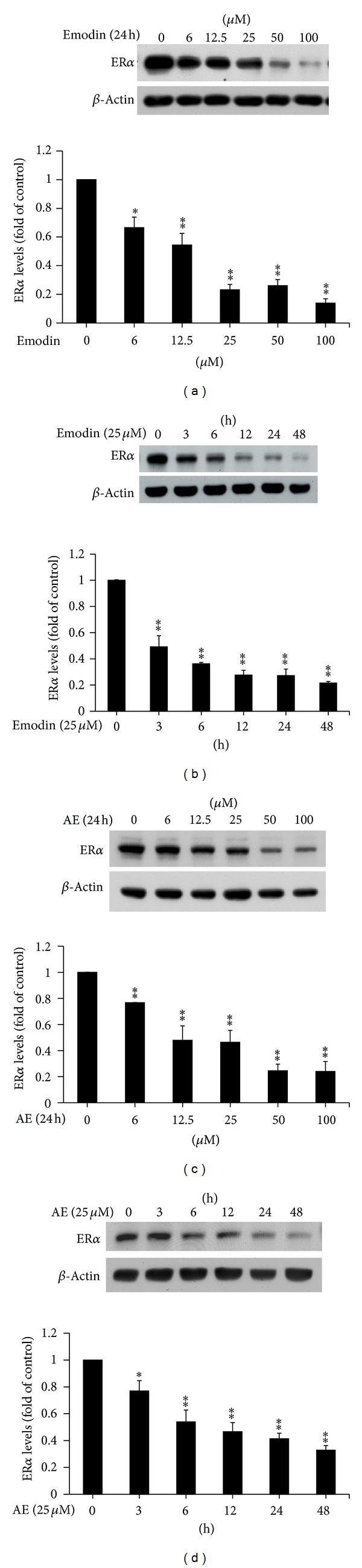
Emodin and aloe-emodin reduce ER*α* protein levels. MCF-7 cells were treated with emodin or aloe-emodin in a dose-dependent manner for 24 h (a, c) and via a time course using 25 *μ*M of the drug (b, d). ER*α* protein levels were detected by western blotting. *β*-Actin was used as an internal control. The quantification of ER*α* levels is presented as fold increase or decrease compared to control levels and was evaluated in three independent experiments. The results were presented as the mean ± SEM. * and ** correspond to *P* < 0.05, and *P* < 0.01, respectively, versus the control group.

 ER*α* activation is triggered by estrogen and promotes the initiation and progression of breast cancer [22, 23]. ER*α* protein levels were detected following treatment with various dosages of emodin or aloe-emodin (0–100 *μ*M, Figures 2(a) and 2(c)) for different time intervals (0–48 h, Figures 2(b) and 2(d)). The quantitative results were provided in the lower panels of the accompanying figures. The data suggest that emodin and aloe-emodin triggered a decrease in ER*α* protein level in a time- and dose-dependent manner.

### 3.3. Emodin and Aloe-Emodin Decrease Both Nuclear and Cytosolic ER*α* and Inhibit ER*α* Activation

**Figure 3:**
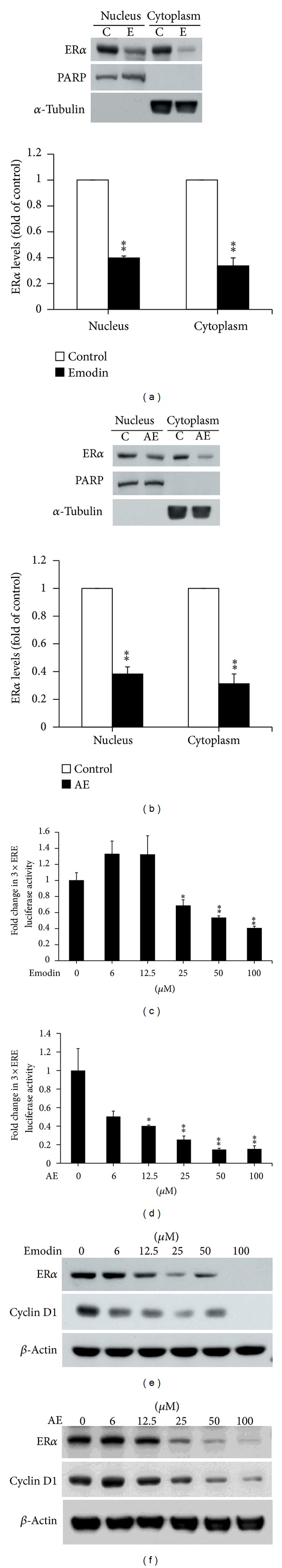
Emodin and aloe-emodin diminish both nuclear and cytoplasmic ER*α* levels and transcriptional activity. MCF-7 cells were treated with 25 *μ*M emodin (a) or aloe-emodin (b) for 24 h prior to cell lysis and protein fractionation as described in Section 2. ER*α* protein was detected by western blotting, and PARP and *α*-tubulin served as markers for the nuclear and cytoplasmic fractions, respectively. The quantification of ER*α* levels was presented as fold increase or decrease relative to controls and was evaluated in three independent experiments. The effects of emodin (c) and aloe-emodin (d) on ER*α* activation were evaluated using a 3 × ERE- (estrogen-responsive element-) containing luciferase reporter assay in MCF-7 cells for 48 h. *β*-galactosidase expression served as the internal control. The data were presented as the fold change compared to control levels (0 *μ*M). The results were presented as the mean ± SEM. * and ** correspond to *P* < 0.05 and *P* < 0.01, respectively, versus control group. MCF-7 cells were treated with emodin (e) or aloe-emodin (f) in a dose-dependent manner for 48 h, and the expressions of ER*α*-regulated protein cyclin D1 were detected by western blotting.

 Because ER*α* protein levels were affected by emodin and aloe-emodin, the activation status of ER*α* was investigated. Fractionation of cellular protein was performed and indicated that emodin and aloe-emodin repressed both the nuclear and cytosolic distribution of ER*α* protein (upper panels in Figures 3(a) and 3(b)). The quantitative results are presented in the lower panels of Figures 3(a) and 3(b). Additionally, cytosolic ER*α* was more sensitive to treatment than nuclear ER*α*. An ER*α* reporter assay was performed in MCF-7 cells. The data showed that aloe-emodin significantly inhibited ER*α*-targeted promoter activity in a dose-dependent manner (Figure 3(d)). Compared to aloe-emodin, emodin in high dosages (25–100 *μ*M) moderately inhibited ER*α* activation, whereas low dosages of emodin (6 and 12.5 *μ*M) tended to increase ER*α* activation. Furthermore, the expressions of ER*α* downstream genes might be affected by those two compounds. Figures 3(e) and 3(f) were the evidence indicating that the protein expression of cyclin D1 which is one of ER*α*-regulated protein was indeed decreased by treatments of 25 *μ*M emodin or 25 *μ*M aloe-emodin for 48 h.

### 3.4. Emodin and Aloe-Emodin Treatment Leads to Decreased ER*α* Protein through Proteasomal Degradation

**Figure 4:**
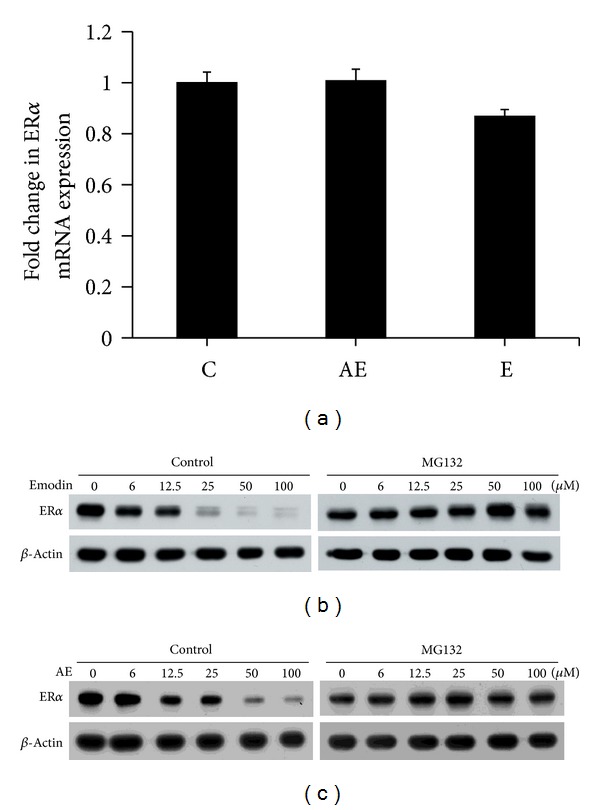
Emodin and aloe-emodin decrease ER*α* protein levels by promoting protein degradation. (a) MCF-7 cells were treated with 25 *μ*M of emodin or aloe-emodin for 24 h prior to quantitative real-time PCR to assess ER*α* mRNA expression. Data were presented as the fold change compared to control levels. The experiments were performed in triplicate, and each condition was replicated four times within each experiment. The results are presented as the mean ± SEM. (b, c) MCF-7 cells were treated with various concentrations of emodin or aloe-emodin in the presence of MG132 (proteasome inhibitor, 5 *μ*M) for 24 h. ER*α* protein levels were detected by western blotting. *β*-Actin was used as an internal control.

 Because ER*α* protein levels were affected by both emodin and aloe-emodin in the previous experiments, the gene expression and protein stability of ER*α* were investigated. First, ER*α* messenger RNA expression was detected by quantitative real-time PCR following treatment. We found that treatments of 25 *μ*M emodin and 25 *μ*M aloe-emodin for 24 h did not have a significant effect compared to the control group (Figure 4(a)). Second, because ubiquitin-proteasome-dependent degradation is the main process involved in ER*α* proteolysis [23], the proteasome inhibitor MG132 was used to prevent ER*α* protein degradation, and drug effects can be observed. The results of this experiment indicated that MG132 could rescue ER*α* degradation after emodin or aloe-emodin treatment (Figures 4(b) and 4(c)). These findings suggest that the decreased ER*α* protein levels observed following emodin or aloe-emodin treatment resulted from proteasome degradation.

### 3.5. Aloe-Emodin Promotes the Dissociation of ER and Heat Shock Protein 90 and Causes ER*α* Ubiquitination

**Figure 5:**
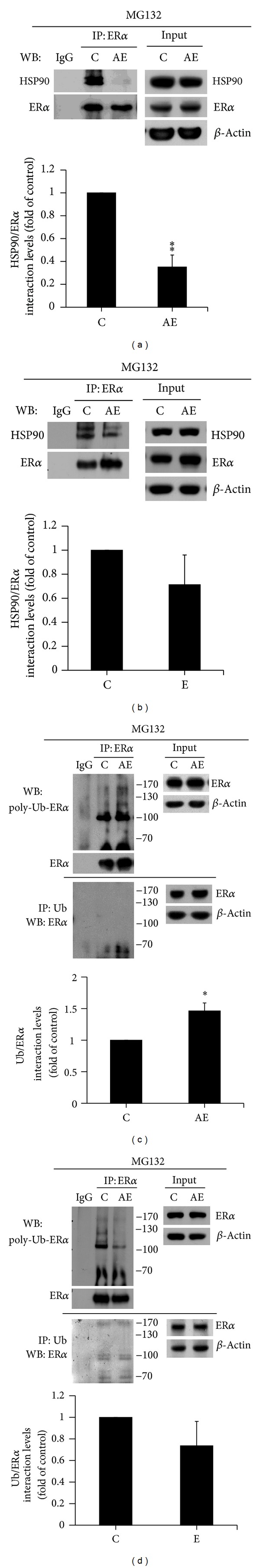
Only aloe-emodin promotes the dissociation of HSP90/ER*α* and subsequent ER*α* ubiquitination. The interaction between ER*α* and HSP90 in MCF-7 cells following 25 *μ*M treatment of aloe-emodin (a) or emodin (b) in the presence of MG132 (5 *μ*M) for 24 h was evaluated by ER*α* immunoprecipitation followed by western blotting for HSP90 with specific antibodies. The ubiquitin-conjugated ER*α* levels in MCF-7 cells following 25 *μ*M treatment of aloe-emodin (c) and emodin (d) in the presence of MG132 (5 *μ*M) were examined by immunoprecipitation with anti-ER*α* or antiubiquitin antibodies followed by western blotting. The lysates were used in the western blotting experiments to indicate protein levels. Immunoprecipitation by IgG served as a negative control. *β*-Actin served as an internal control for western blotting. The quantitative data were obtained from the results of three times immunoprecipitation experiments and presented as fold change of control. The quantitative graphs were presented as the mean ± SEM. * and ** correspond to *P* < 0.05 and *P* < 0.01, respectively, versus the control group.

ER*α* is processed by ubiquitination after disassociating from heat shock protein 90 (HSP90) [24]. Using immunoprecipitation in the presence of MG132 to prevent protein degradation, we found that aloe-emodin apparently blocked the protein interaction between ER*α* and HSP90 (Figure 5(a)); however, the effect induced by emodin was not as significant (Figure 5(b)). The quantitative graphs were provided in the lower panels of accompanying figures. Subsequently, ER*α* ubiquitination following drug treatment was investigated by detecting the levels of ubiquitinated ER*α* in drug-treated cell extracts following MG132 administration. The data indicated that aloe-emodin treatment enhanced ubiquitin-conjugated ER*α* levels, and protein degradation was prevented by MG132 treatment regardless of whether ER*α* or ubiquitin was immunoprecipitated first (Figure 5(c)). Although emodin slightly promoted ER*α*/HSP90 dissociation (Figure 5(b)), no increase in ER*α* ubiquitination was observed following emodin treatment (Figure 5(d)). The related quantitative results were shown in the lower panels. It suggests that ubiquitination was not required for emodin-induced ER*α* degradation by proteasome. These results indicate that aloe-emodin specifically reduces ER*α* protein levels by promoting ER*α* ubiquitination, which results in proteasome-dependent degradation.

### 3.6. Comparison of Emodin/Aloe-Emodin and Estrogen on ER*α* Behaviors

**Figure 6:**
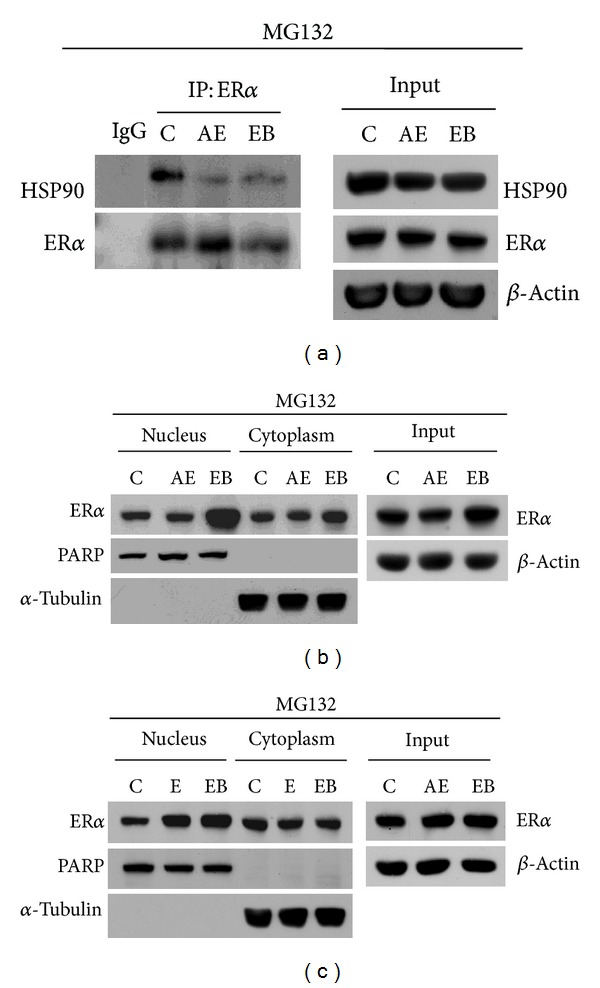
Effects of aloe-emodin and emodin on ER*α* subcellular distribution in comparison to EB. (a, b) MCF-7 cells were treated with aloe-emodin (25 *μ*M) or estradiol benzoate ((EB) 10 nM) in the presence of MG132 (5 *μ*M) for 24 h. The interaction between HSP90 and ER*α* was evaluated by ER*α* immunoprecipitation followed by western blotting of HSP90 with specific antibodies. Immunoprecipitation by IgG served as a negative control. The fractionation of cellular proteins was performed, and ER*α* protein was detected by western blotting. (c) MCF-7 cells were treated with emodin (25 *μ*M) or EB (10 nM) in the presence of MG132 (5 *μ*M) for 24 h. The fractionation of cellular proteins was performed, and ER*α* protein was detected by western blotting. PARP and *α*-tubulin served as markers for the nuclear and cytoplasmic fractions, respectively. The lysates were used in the western blotting experiments to indicate protein levels. *β*-Actin served as an internal control for western blotting.

 Ligand binding by estrogen or estrogen-like molecules promotes ER*α* transactivation, during which ER*α* first dissociates from HSP90 in the cytoplasm and then translocates into the nucleus [8]. Because the effects of aloe-emodin and emodin on the ER*α* ubiquitination process are distinct (Figure 5), it is of interest to understand how the effects of these two compounds compare to those of estrogen to mediate ER*α* behavior. Immunoprecipitation experiment results indicate that aloe-emodin had similar effects to synthetic estrogen (estradiol benzoate (EB)) on ER*α*/HSP90 dissociation (Figure 6(a)), whereas emodin did not (Figure 5(b)). However, in contrast to EB treatment, aloe-emodin treatment led to a decrease in the nuclear and cytoplasmic levels of ER*α* protein. Furthermore, the decrease in ER*α* protein levels following aloe-emodin treatment was blocked by MG132, suggesting that aloe-emodin promotes ER*α* degradation (Figure 6(b)). Comparing the data in Figure 3(b), we suggest that aloe-emodin preferentially induces ER*α* degradation in the cytoplasm of breast cancer cells. Interestingly, nuclear ER*α* was increased by emodin treatment, and protein degradation was prevented by MG132 (Figure 6(c)), suggesting that although emodin induces ER*α* degradation in both the nucleus and cytoplasm (compared to Figure 3(a)), it seems that emodin is able to promote ER*α* translocation into the nucleus, which is in contrast to the actions of aloe-emodin. This might explain why low concentrations of emodin showed slight stimulation in the ER*α* reporter assay (Figure 3(c)). Taken together, these results demonstrate that aloe-emodin induces the dissociation of ER*α*/HSP90 and subsequently promotes ER*α* ubiquitin-proteasome-dependent degradation in the cytoplasm, thereby inhibiting ER*α* translocation into nucleus, where ER*α* would otherwise be activated as a positive modulator.

## 4. Discussion

Breast cancer is a common malignancy in women, and estrogen plays an important role in early cancer development [25]. Estrogen, which usually denotes 17*β*-estradiol (E2), binds to its primary receptor ER*α* and stimulates ER*α* transcriptional activity to regulate downstream gene expression and cell growth [26, 27]. Tamoxifen is designed to interfere with E2 binding and thus block ER*α* transcriptional activity to treat ER*α*-dependent diseases such as breast cancer [28]. However, alternative compounds that are safer and associated with fewer side effects than Tamoxifen are desired. Numerous studies suggest that phytoestrogens possess organ-specific estrogenic and antiestrogenic effects [29]. Phytoestrogens mainly consist of isoflavones, such as genistein and daidzein, and are used in the treatment of menopausal symptoms as well as breast cancer [30, 31]. In this study, we provide evidence indicating that two phytoestrogens, emodin and aloe-emodin, might significantly inhibit the proliferation of ER*α*-positive breast cancer cells through ER*α* degradation. Although both drugs had dose-responsive effects on inhibiting proliferation of breast cancer cells, low doses (25 *μ*M or less) of aloe-emodin could not affect proliferation of ER*α*-negative cells (Figure 1(f)). Therefore, 25 *μ*M of drugs were utilized in other experiments (Figure 3 to Figure 6). Interestingly, both compounds had inhibitory effects on ER*α*, but they utilized different inhibitory mechanisms. Aloe-emodin inhibited ER*α* activation through HSP90/ER*α* dissociation and ubiquitin-dependent degradation, whereas emodin did not share the same molecular pathway. Therefore, these findings illustrate that emodin and aloe-emodin might serve as estrogen receptor modulators with different molecular mechanisms. The therapeutic application of these two compounds should be investigated further in the future.

Anthraquinones are phytoestrogens that have been demonstrated to possess anticancer properties through the inhibition of cell proliferation, the induction of apoptosis, and the prevention of metastasis [1]. The effects of the anthraquinone derivatives emodin and aloe-emodin have been extensively investigated in several cancer types through studies on their signaling targets. Emodin has been reported to sensitize Her2/neu-overexpressing lung cancer cells to chemotherapeutic treatments and to suppress Her2/neu-overexpressing breast cancer growth by inhibiting tyrosine kinase activity [32–34]. Notably, emodin has been shown to downregulate androgen receptor (AR) and inhibit prostate cancer growth [5], suggesting that there is a connection between anthraquinone derivatives and steroid receptor in hormone-related cancers. Although the estrogenic ability of emodin is higher than that of aloe-emodin, as determined by ER*α* binding studies, the antiproliferation effect of aloe-emodin is more efficient than that of emodin in breast cancer cells [7]. Kang et al. also showed that the cytotoxicity of aloe-emodin is higher in ER*α*-positive cells than in ER*α*-negative cells [7]. These observations suggest that aloe-emodin might be a stronger inhibitor of ER*α*-positive cancer cell growth than emodin.

 The goal of this study was to investigate the differences between emodin and aloe-emodin in their efficiency and mechanism of blocking breast cancer cell growth. Our data showed that aloe-emodin is a more potent growth inhibitor than emodin and has a unique mechanism for the growth inhibition of breast cancer cells. MCF-7 cell growth was abolished following treatment with 12.5 *μ*M aloe-emodin for 6 days, and only 50% of cells survived at 25 *μ*M aloe-emodin treatment for 4 days (Figure 1(b)). Although a previous study suggested that the IC_50_ of aloe-emodin is 12.6 ± 0.83 *μ*g/mL (approximately 46 *μ*M) on MCF-7 cells [7], the IC_50_ of aloe-emodin in our system was only approximately 25 *μ*M (Figure 1(d)). With regard to side effects on normal cells, the previous report indicated that 25 *μ*M aloe-emodin is a more significant proliferation inhibitor in human skin epidermoid carcinoma cells than in noncancerous cells [35]. However, aloe-emodin did not significantly affect the proliferation of MDA-MB-453 ER*α*-negative cells (Figure 1(f)). The overexpression of ER*α* in MCF-7 cells increased the sensitivity to aloe-emodin treatment (Figure 1(h)). These results are consistent with previous findings that aloe-emodin has a higher cytotoxic potential to MCF-7 (ER*α*-positive) cells than to MDA-MB-231 (ER*α*-negative) cells [7]. A low dosage (10 *μ*M) of aloe-emodin [6] showed that both cell growth (Figures 1(b) and 1(d)) and ER*α* activation (Figure 3(d)) were significantly repressed by aloe-emodin in a dose-dependent manner, even at low dosages (6 *μ*M). Additionally, aloe-emodin treatment reduced ER*α* protein levels not only in total lysates (Figures 2(c) and 2(d)) but also in nuclear and cytoplasmic fractions (Figure 3(b)). In the nuclear and cytoplasmic fractions, the impact of aloe-emodin treatment on cytoplasmic ER*α* levels seems stronger than that on nuclear ER*α* levels (Figure 3(b)). As shown in Figures 2 and 4, we conclude that the decrease in ER*α* levels induced by aloe-emodin was due to protein degradation. This correlates with ER*α* proteasome-dependent degradation because of the observed elevation in ubiquitin-conjugated levels (Figure 5(c)). Interestingly, the dissociation of ER*α* and HSP90 was increased by aloe-emodin treatment (Figure 5(a)), and ER*α* released from HSP90 protection was subjected to ubiquitination for degradation rather than translocation to the nucleus for activation (Figure 6(b)). These phenomena are similar to the actions of antiestrogens such as ICI 164384, ICI 182780, and RU 58668, which may induce ER*α* degradation and result in rapid turnover [24]. As a ligand with an inhibitory effect on ER*α*, we suggest that aloe-emodin might qualify as an estrogen receptor modulator to negatively regulate ER*α* activity and inhibit breast cancer cell growth.

The effect of emodin was also investigated in this study. Although the inhibitory effects of emodin on breast cancer growth and ER*α* activation were similar to those of aloe-emodin, the molecular mechanism of emodin inhibition was unexpectedly distinct. In Figures 1(a) and 1(c), the inhibitory potency of emodin on MCF-7 cell growth was lower than that of aloe-emodin (Figures 1(b) and 1(d)). Interestingly, emodin significantly suppressed cell proliferation of the ER*α*-negative cell line MDA-MB-453 (Figure 1(e)). Because emodin may act as a tyrosine kinase inhibitor by inhibiting the binding of Her2/neu and HSP90, thereby depleting Her2/neu via proteasomal degradation [36], it is possible that emodin might differ in its effect on the growth of MDA-MB-453 cells (which are characterized by Her2 overexpression) in ER*α*-independent regulation in comparison to aloe-emodin. Like aloe-emodin, emodin targeted ER*α* by proteasomal degradation (Figures 2 and 4); however, the degradation pathway was independent of HSP90/ER*α* dissociation and ubiquitination (Figures 5(b) and 5(d)). Although the ubiquitin-proteasome degradation is an important process for modulation of many proteins, recent studies demonstrate that the proteasome-dependent protein degradation possibly goes through ubiquitination-independent pathway [37, 38]. Previous reports indicate that the estrogenic activity of emodin is higher than that of aloe-emodin [6, 7]. Our ERE reporter assay results showed that emodin induced the inhibition of  ER*α* activation only at high dosages (over 25 *μ*M, Figure 3(c)), whereas the aloe-emodin data showed inhibitory effects starting at low dosages (6 *μ*M, Figure 3(d)). Notably, emodin treatment led to a slight increase in ER*α* activation at 6 and 12.5 *μ*M, which differed from the aloe-emodin treatment results (Figure 3(c)). Unlike aloe-emodin, emodin treatment elevated nuclear ER*α* protein levels in the presence of  MG132, similar to the effects of estradiol benzoate (EB) treatment (Figure 6(c)). Taken together with the data in Figure 3(a), we suggest that emodin, using a distinct mechanism from aloe-emodin, might cause ER*α* shuttling into the nucleus and subsequently promote nuclear ER*α* degradation through ubiquitination-independent pathway, whereas cytoplasmic ER*α* is less affected. This hypothesis is similar to previous studies indicating that the chemical structure of the ligand directly affects the nuclear fate and protein turnover rate of ER*α* independently of transcriptional regulation [39]. These observations suggest that the essential difference between emodin and aloe-emodin lies in the mechanism of ER*α* regulation and Her2 inhibition.

In conclusion, we provide evidence showing that the anticancer mechanisms of the anthraquinone derivatives emodin and aloe-emodin are mediated via an ER*α*-dependent pathway in breast cancer cells. Although emodin and aloe-emodin display distinct differences in efficiency and ER*α* activation mechanism on breast cancer cell growth, both compounds are potential selectively therapeutic treatments for breast cancer.

## Abbreviations

ER*α*: Estrogen receptor *α*
HSP90: Heat shock protein 90
AE: Aloe-emodin
E: Emodin.
